# Pyrethroid resistance in southern African *Anopheles funestus *extends to Likoma Island in Lake Malawi

**DOI:** 10.1186/1756-3305-3-122

**Published:** 2010-12-31

**Authors:** RH Hunt, M Edwardes, M Coetzee

**Affiliations:** 1 Malaria Entomology Research Unit, School of Pathology, Faculty of Health Sciences, University of the Witwatersrand, Johannesburg, South Africa; 2 Vector Control Reference Unit, National Institute for Communicable Diseases of the National Health Laboratory Service, Private Bag X4, Sandringham 2131, South Africa; 3 Bayer Environmental Science, P.O. Box 143, Isando, 1600, South Africa

## Abstract

**Background:**

A mosquito survey was carried out on the island of Likoma in Lake Malawi with a view to collecting baseline data to determine the feasibility of implementing an integrated malaria vector control programme. No vector control interventions are currently being applied on the island apart from the sporadic use of treated and untreated bed nets.

**Results:**

Large numbers of *Anopheles funestus *were found resting inside houses. WHO susceptibility tests were carried out on wild caught females and 1-5 day old F-1 female progeny. Wild caught females were tested on deltamethrin (77.8% mortality) and bendiocarb (56.4% mortality). Female progeny were tested on deltamethrin (41.4% mortality), permethrin (40.4%), bendiocarb (52.5%), propoxur (7.4%), malathion, fenitrothion, DDT, dieldrin (all 100%) and pirimiphos-methyl (98.9%). The malaria parasite rate was 4.9%. A small number of *Anopheles arabiensis *were also collected.

**Conclusion:**

This locality is 1,500 km north of the currently known distribution of pyrethroid resistant *An. funestus *in southern Africa. The susceptibility results mirror those found in southern Mozambique and South African populations, but are markedly different to *An. funestus *populations in Uganda, indicating that the Malawi resistance has spread from the south.

## Background

*Anopheles funestus *is the major malaria vector in southern Africa. Early records of its involvement in malaria transmission give *Plasmodium falciparum *parasite rates as high as 22% in South Africa [1]. More recently, in Tanzania 11% infection rate was recorded [2] and 5% in southern Mozambique [3].

South Africa eradicated *An. funestus *in the 1950's when an extensive indoor residual spraying (IRS) campaign using DDT was rolled out. In the next 50 years, this vector species was recorded only once during a small malaria outbreak in the northern part of the country [4]. In 1999/2000, however, South Africa experienced its worst malaria outbreak since the introduction of IRS in the 1950's and *An. funestus *was found once again in northern KwaZulu/Natal, just south of Mozambique [5,6]. The *P. falciparum *parasite rate in *An. funestus *was 5.4% and the mosquitoes were found to be resistant to both pyrethroids and carbamates.

Subsequent research in southern Mozambique showed that the insecticide resistant population of *An. funestus *extended north of the capital, Maputo [7-9]. Most recently, resistance was found in *An. funestus *from Chokwe [10], approximately 200 km north of the capital, where previously this population was found to be susceptible [8].

The present study provides evidence of insecticide resistance in *An. funestus *from an island in Lake Malawi that is considerably further north than any previous records of resistance.

## Materials and methods

### Study site

**Figure 1:**
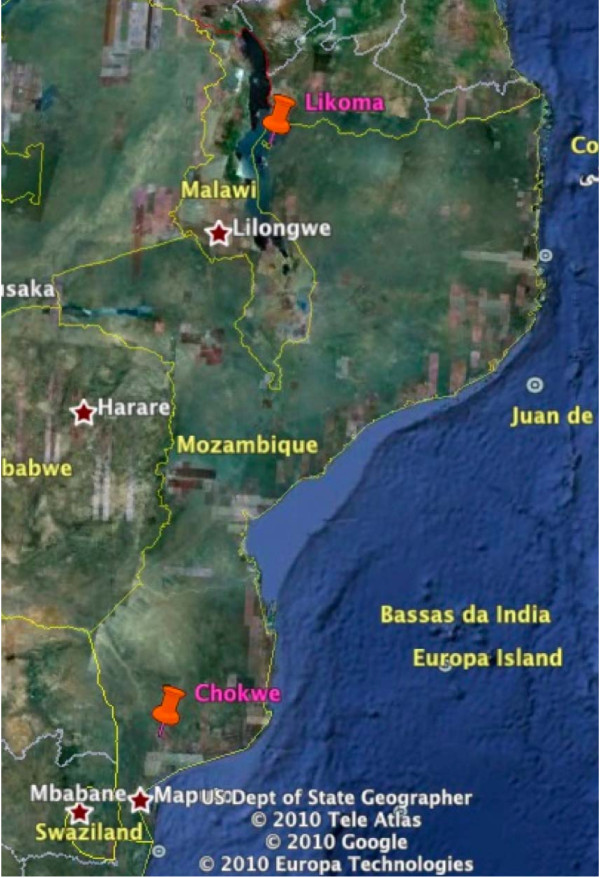
**Map of Malawi and Mozambique showing Likoma island in the north and the currently known limit of pyrethroid resistant *An. funestus *at Chokwe in the south of Mozambique **[10]**, approximately 1,500 km apart**.

The mosquito survey was carried out on Likoma Island in Lake Malawi (12°04'S, 34°44'E) from 10 - 14 May 2010 (Figure 1). The island is a series of outcrops and the housing on the island consists mainly of scattered homesteads with residents engaged in fishing and small-scale subsistence farming. Many houses were searched for mosquitoes mostly without success, but a substantial *An. funestus *population was found in a few houses close to a small area being used for rice cultivation.

### Mosquito collections

Mosquitoes were collected resting inside houses using a hand aspirator. Some samples were used immediately for WHO susceptibility tests while others were packaged and returned to Johannesburg where egg batches were obtained and larvae reared through to F-1 adults.

### Laboratory processing

Species identification was carried out using the methods of Koekemoer et al. [11] for the *An. funestus *group and Scott et al. [12] for the *An. gambiae *complex.

Wild females were screened for malaria parasite infection using ELISA [13].

Insecticide susceptibility tests were carried out using the WHO [14] standard test kits and treated papers from the WHO Collaborating Centre in Penang, Malaysia. The insecticides tested and their discriminating doses are given in Table 1 and 2.

**Table 1:** Insecticide susceptibility tests carried out on An. funestus females on Likoma Island.

	Total	No. dead	No. alive	% Mortality 24-hr post-exposure
0.05% Deltamethrin	53	42	11	79.2

0.1% Bendiocarb	27	16	11	59.2

Control	31	2	29	6.5

**Table 2:** Insecticide susceptibility tests carried out on 1-5 day old female progeny of An. funestus from Likoma Island.

	Total	No. dead	No. alive	% Mortality
0.05% Deltamethrin	174	72	102	41.4

0.75% Permethrin	146	59	87	40.4

0.1% Bendiocarb	141	74	67	52.5

0.1% Propoxur	54	4	50	7.4

5% Malathion	126	126	0	100

1% Fenitrothion	103	103	0	100

0.9% Pirimiphos methyl	99	98	1	98.9

4% DDT	155	155	0	100

4% Dieldrin	137	137	0	100

Controls	137	0	137	0

## Results

One hundred and eleven wild *An. funestus *females of unknown age were tested for insecticide resistance under field conditions with no temperature or humidity control. A total of 6 *An. gambiae *complex females and over 120 females and ± plusorminus90 males of *An. funestus*, together with a small collection of *An. gambiae *larvae, were packaged and transported back to the laboratory in Johannesburg.

A total of 223 *An. funestus *were subjected to molecular assays including all the wild adults used in the susceptibility tests (n = 111) as well as the live females brought back to the laboratory for egg laying (n = 112). 97.3% were successfully identified as *An. funestus s.s*. (five specimens did not amplify a PCR product and one specimen was identified as *An. funestus-like*). All males and females of the *An. gambiae *complex (wild adults and adults reared from larvae, n = 89) were identified as *An. arabiensis*.

Of the 81 wild *An. funestus *females tested for parasite infection, 4.9% were positive for *P. falciparum *using the ELISA method.

The results of the first insecticide susceptibility tests, carried out on the island using wild female *An. funestus *of unknown age, are given in Table 1. Since the controls gave >5% mortality, Abbott's formula [14] was used to correct the results, giving 77.8% mortality on deltamethrin and 56.4% on bendiocarb. The papers used in the field were tested in the laboratory using a susceptible *An. gambiae *colony and gave 100% mortality for all samples and replicates (n = 100 for each insecticide).

The second round of insecticide susceptibility tests was carried out in the laboratory at 25°C and 85% RH using 1-5 day old *An. funestus *females pooled from approximately 120 egg batches. Nine different insecticides from all four classes were tested and the results are given in Table 2.

Unfortunately, the *An. arabiensis *sample reared from larvae was too small (n = 42 females) to carry out meaningful susceptibility tests.

## Discussion

The marked difference between the deltamethrin susceptibility tests carried out on wild females in the field and those on the laboratory reared, 1-5 day old F-1 progeny (p <0.005), can be explained in two ways. One, high temperatures are known to affect the survival of mosquitoes exposed to insecticides [15] and this may account for the high mortality in the field samples. Two, *An. funestus *susceptibility to this sub-class of pyrethroids may be age dependent [16]. Since the survey was carried out in May towards the end of the transmission season, it is likely that the wild-caught females tested in the field were an aging population and were therefore more susceptible to the insecticides. However, Hunt et al. [16] also report that blood fed, mated, females did not show any decrease in resistance over time, and aging wild populations would all be mated and have taken numerous blood meals.

It is clear from the susceptibility results that a resistance management strategy will have to be devised and implemented in order to control malaria on the island. If pyrethroid treated bed nets are to be distributed widely on Likoma Island, then IRS must be carried out simultaneously with an organophosphate or DDT in order to manage the resistance. Carbamates are unfortunately not an option with such a high frequency of survival. The *An. funestus *population is fully susceptible to DDT, which raises the possibility of using DDT for IRS perhaps in a rotation with one of the organophosphates.

**Figure 2:**
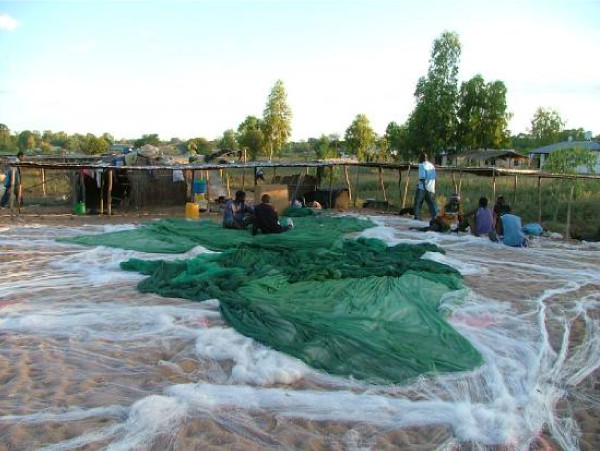
**Fishermen on Likoma drying their nets**. The green nets are bed nets that have been modified for fishing.

There is already extensive use of bed nets on the island with an assortment of treated and untreated nets, old and new, damaged and intact. There is also obvious variation in usage. Frequently, nets were present in the house but not being used. If a combination of bed nets and IRS is under consideration, an important component of such a strategy must be education and monitoring of net use. When the mosquito populations decrease, either due to seasonal change or in response to control measures, many people will stop using the nets. It is also a reality that in a community where livelihood depends on fishing, some nets will be used for this purpose (Figure 2).

The most worrying aspect of this survey is the discovery of pyrethroid and carbamate resistance in the *An. funestus *population approximately 1,500 km north (Figure 1) of its current known distribution at Chokwe in southern Mozambique [10]. The report by Casimiro *et al*. [9] on samples collected from central Mozambique in 2006 showed that *An. funestus *had >95% mortality to pyrethroids and carbamates. The WHO criteria recommend that this percentage of susceptibility requires further investigation, but operationally it is unlikely that a control programme would change its policy based on this frequency of resistance/susceptibility.

Likoma Island in Lake Malawi is just a few kilometres away from Mozambique and presumably the mosquitoes are either blown over by the wind or brought on boats that ply their trade between the island and the mainland. One must assume, therefore, that the *An. funestus *population in northern Mozambique is also resistant and this has serious implications for current malaria control efforts being undertaken in this region. Since both pyrethroid and carbamate resistance has been found in the Likoma population, mirroring the resistance found in more southerly populations, it can be assumed that the resistance is spreading northwards through the *An. funestus *populations through gene flow, and not arising as separate genetic mutation events. There are no obvious geographical barriers to gene flow in this region of southern Africa and presumably we can expect the resistance to spread northwards into southern Tanzania and westwards into Zambia and Zimbabwe. The recently reported resistance in *An. funestus *from Uganda [17] is obviously different to that observed in southern African populations, based on both susceptibility tests and molecular characterization of the P450 genes [5,16,18,19]. In Uganda, both pyrethroid and DDT resistance was found, with full susceptibility to carbamates, suggesting that this resistance has arisen independently under different selection pressures as suggested by Morgan et al. [10] based on their molecular data.

This paper highlights the seriousness of the rapid spread of insecticide resistance in *An. funestus *in southern Africa and the urgent need for resistance management strategies within malaria vector control programmes within the region.

## Competing interests

The authors declare that they have no competing interests.

## Authors' contributions

All authors had input into the conception of the project, data analysis and drafting of the manuscript.
